# Molecular Analysis of Rabies Virus Using RNA Extracted from Used Lateral Flow Devices

**DOI:** 10.1128/jcm.01543-22

**Published:** 2023-02-22

**Authors:** Jaira D. Mauhay, Nobuo Saito, Kazunori Kimitsuki, Milagros R. Mananggit, Jeffrey L. Cruz, Maria G. Lagayan, Alyssa M. Garcia, Patricia M. Lacanilao, Kentaro Yamada, Mariko Saito-Obata, Daria L. Manalo, Catalino S. Demetria, Beatriz P. Quiambao, Akira Nishizono

**Affiliations:** a Department of Microbiology, Faculty of Medicine, Oita University, Yufu, Oita, Japan; b School of Tropical Medicine and Global Health, Nagasaki University, Nagasaki, Nagasaki, Japan; c Regional Animal Disease Diagnostic Laboratory, Department of Agriculture Field Office III, San Fernando, Pampanga, Philippines; d Department of Agriculture, Bureau of Animal Industry, Quezon, National Capital Region, Philippines; e Laboratory of Veterinary Public Health, Department of Veterinary Medical Science, Faculty of Agriculture, University of Miyazaki, Miyazaki, Miyazaki, Japan; f Tohoku University Graduate School of Medicine, Sendai, Miyagi, Japan; g Research Institute for Tropical Medicine, Muntinlupa, National Capital Region, Philippines; h Research Center for Global and Local Infectious Diseases, Faculty of Medicine, Oita University, Yufu, Oita, Japan; Jockey Club College of Veterinary Medicine

**Keywords:** Philippines, lateral flow devices, molecular methods, rabies

## Abstract

Molecular analysis of rabies virus can provide accurate diagnosis and information on its genetic diversity. The transportation of rabies brain samples from remote areas to a central laboratory is challenging owing to biohazard risks and decomposability. We investigated the utility of used lateral flow devices (LFDs) for subsequent molecular analysis and assessed the necessary storage temperatures. Using RNA extracted from used LFD strips, we performed conventional reverse transcription-PCR (RT-PCR) using an LN34 primer set to amplify short fragments (165 bp) for rabies virus detection and the P1-304 primer set to amplify long fragments of the entire N gene amplicon (1,506 bp) for phylogenetic analysis. Among 71 used LFDs stored in a refrigerator and 64 used LFDs stored at room temperature, the LN34 assay showed high sensitivities (96.2% and 100%, respectively) for the diagnosis of rabies, regardless of the storage temperature. A significant reduction in the sensitivity of rabies diagnosis was observed when using the P1-304 primer set for used LFDs stored at room temperature compared to those stored at refrigeration temperature (20.9% versus 100%; *P* < 0.05). Subsequent sequencing and phylogenetic analysis were successfully performed using the amplicons generated by the P1-304 RT-PCR assays. Used LFDs are thus promising resources for rabies virus RNA detection and sequence analysis. Virus detection via RT-PCR, amplifying a short fragment, was possible regardless of the storage temperature of the used LFDs. However, refrigerated storage is recommended for RT-PCR amplification of long fragments for phylogenetic analysis.

## INTRODUCTION

Understanding the transmission dynamics of rabies virus with genome sequence analysis can provide valuable information for establishing an effective control strategy against the virus. However, molecular epidemiological surveillance has not been consistently performed in low- and middle-income countries. This is because while a standard rabies diagnostic test, the direct fluorescent-antibody test (DFAT), can be performed in regional laboratories, only some central research laboratories can perform molecular analysis, including sequencing. Rabies clinical samples are mainly from the brain and are biohazardous. As such, strict biosafety handling and a cold environment are required to avoid autolysis. Therefore, sample transportation often poses significant cost and logistic challenges, particularly in island countries such as the Philippines. In areas where rabies is endemic, this constitutes a bottleneck in performing genome sequencing of the virus (1).

Recent evidence has indicated the usefulness of filter paper for molecular analysis because it enables the easy storage and shipment of biological materials such as blood, saliva, or organ tissues (2–4). Some studies on rabies virus have reported high performances of molecular tests using brain tissue dried on filter papers (5–8). Furthermore, various studies have indicated that DNA or RNA of pathogenic microorganisms such as *Plasmodium* spp., dengue virus, chikungunya virus, and Zika virus can be extracted from used lateral flow devices (LFDs) for molecular detection and genotyping (9–12). LFDs have the strong advantage of being used for point-of-care testing. The usefulness and accuracy of LFDs for the diagnosis of rabies in animals have been shown in recent studies in the Philippines (13, 14). Rabies animal surveillance would be strengthened if LFDs were used not only for on-site diagnosis but also for subsequent molecular analysis. However, there are few reports on rabies virus investigating the utility of RNA extracted from LFDs (15–17). Léchenne et al. applied reverse transcription-PCR (RT-PCR) and molecular sequencing techniques using filter paper from Anigen rapid rabies test kits (Bionote, Inc., Hwaseong, South Korea) (15, 16). Among 51 samples with positive LFDs, RT-quantitative PCR (RT-qPCR) using RNA extracted from the LFDs detected 44 (86.3%) positive samples. They obtained sequence data from 13 out of 14 samples (16). This methodology is sound, but the main limitation of that study was the sample size for sequencing. Concerns about RNA stability during the storage of used LFDs at room temperature have also been raised (5). In the present study, we evaluated the utility and diagnostic accuracies of RT-PCR using RNA extracted from used LFDs after the diagnostic test compared with those of the gold-standard test (DFAT). We also evaluated the appropriate storage temperature for used LFDs for subsequent molecular analysis.

## MATERIALS AND METHODS

This analysis was implemented under an existing study to evaluate LFDs for postmortem rabies diagnosis in animals (13, 14, 18). This study was performed at the Regional Animal Diagnostic Laboratory in Central Luzon, Philippines. This laboratory routinely receives decapitated animal heads for rabies confirmatory testing, namely, DFATs. When this laboratory received samples, brain specimens for LFD testing were collected by a simple sampling methodology using a drinking straw (14, 15, 19, 20). The collected brain samples were homogenized in a BioMasher II instrument (Nippi, Tokyo, Japan) with approximately 400 to 500 μL of the assay buffer provided by the LFD kit manufacturer (rabies antigen test kits, lot no. 1904; Adtec Co., Ltd., Oita, Japan) (14). Next, 100 μL of the suspension was added to the sample well of the test kit. The results were read 15 min after applying the sample. Brain samples for the DFAT were separately collected after opening the skull from the hippocampus, brain stem, and cerebellum (13). This laboratory does not perform DFATs when decomposed brain samples are submitted because of the effect of degradation on the tests. We excluded the samples for which this laboratory did not perform DFATs.

### Study procedure.

**FIG 1:**
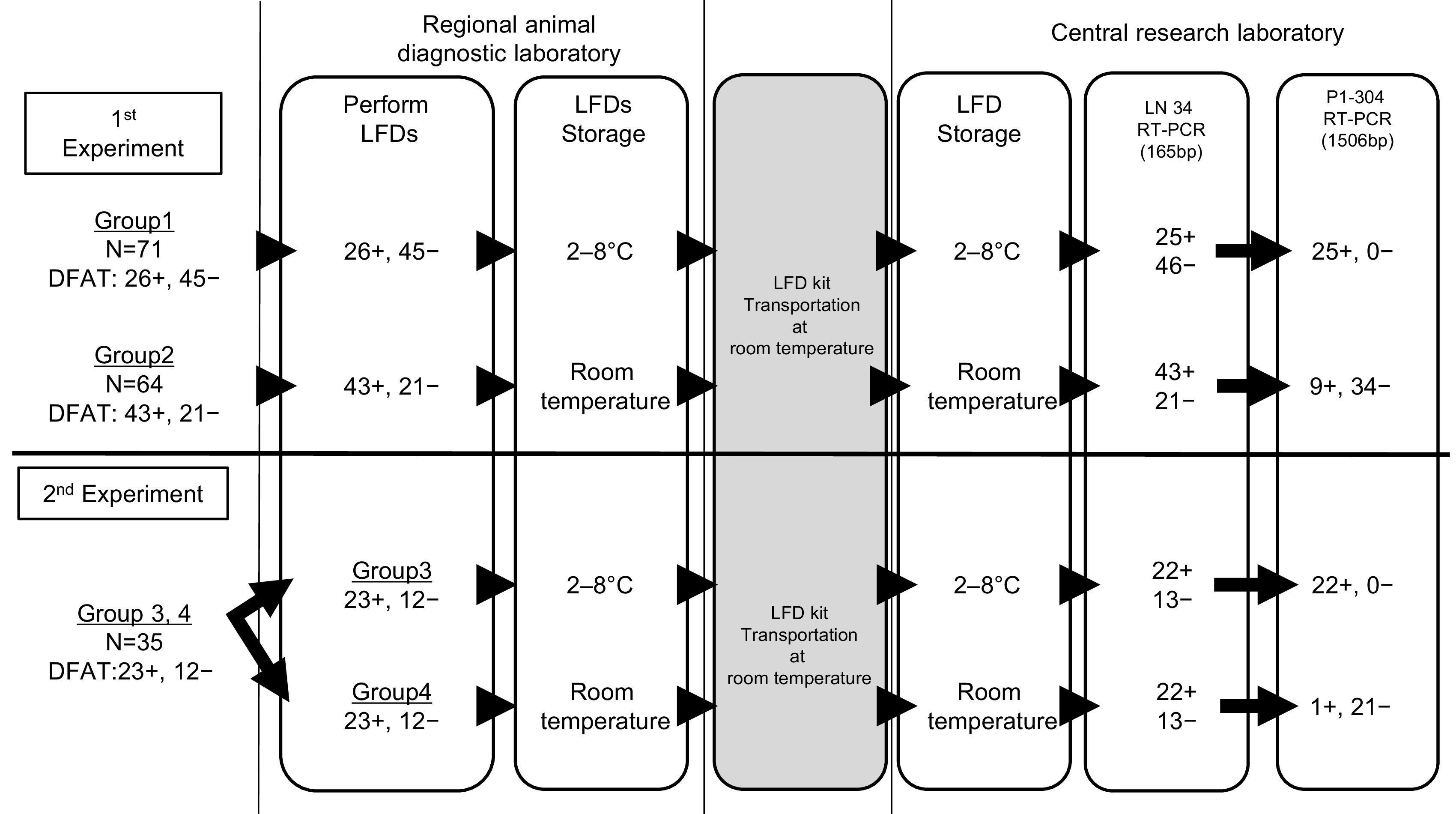
Study flow and results of on-site DFAT, on-site LFD, conventional LN34 RT-PCR, and conventional P1-304 RT-PCR. +, positive result; −, negative result.

The study procedure following LFD tests and DFATs at the regional animal diagnostic laboratory up to molecular analysis at the central laboratory is shown in Fig. 1. First, we collected used LFDs after diagnostic testing and stored them in temperature-controlled refrigerators at 2°C to 8°C (group 1). After the target number of used LFDs was reached, we collected additional used LFDs and stored them at room temperature to analyze the temperature stability of used LFDs for RT-PCR (group 2). For further analysis as a second experiment, we applied one brain specimen to two LFDs. Next, we stored used LFDs at room temperature (group 3) or refrigerator temperature (group 4). The room temperature range in the storage room was between 15°C and 30°C. All LFDs were transferred to the central research laboratory in Metro Manila at room temperature, with the duration of transportation being 3 to 4 h. We stored the used LFDs in the central laboratory under the same conditions as those prevalent in the regional laboratory until RNA extraction.

### RNA extraction from LFD strips.

**FIG 2:**
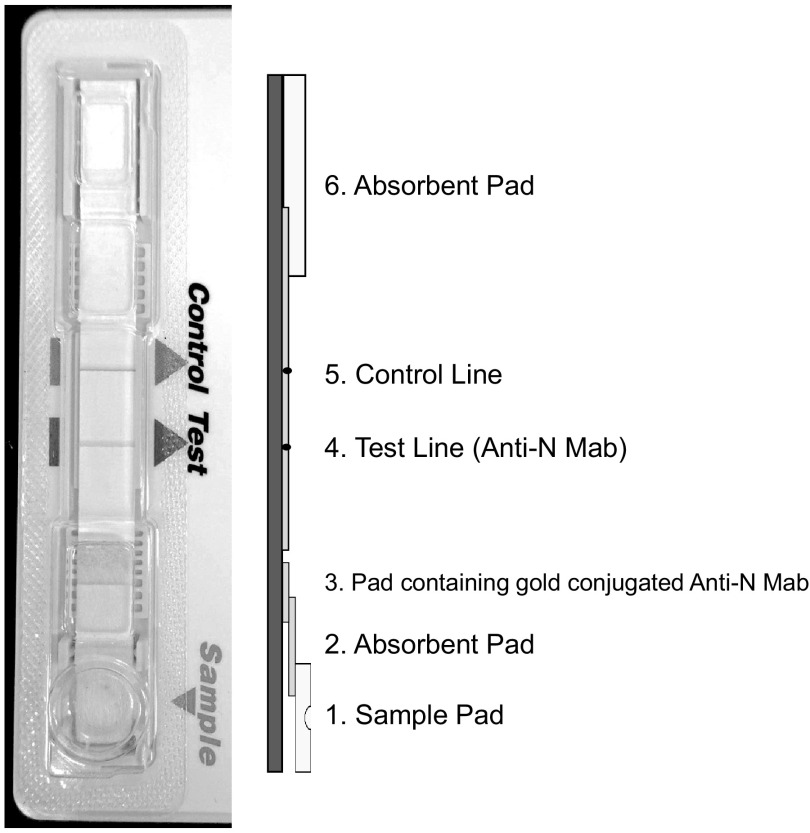
Illustration of a used rabies antigen LFD and lateral sketch of the internal sections of the cassette. The pad containing gold-conjugated anti-N monoclonal antibody (Mab) (3) and a portion of the absorbent pad at the left (6) were used for viral RNA recovery.

In the central laboratory, we removed the plastic cover (using scissors or forceps) from the LFD cassettes and detached the nitrocellulose strip from the cassettes. There are four absorbent papers in the strip. We used two of the papers for RNA extraction (Fig. 2). These two absorbent papers were transferred to a microcentrifuge tube containing 1 mL Isogen II (Nippon Gene Co., Ltd., Toyama, Japan) and incubated the tube for an hour. Next, RNA was extracted according to the manufacturer’s manual and stored at −80°C until further use (see Fig. S1 in the supplemental material).

### RT-PCR assay with the LN34 primer set for virus detection.

First, we evaluated the sensitivity and specificity of conventional RT-PCR using an LN34 primer set for viral RNA detection with the DFAT as the reference test. The LN34 assay was used for the amplification of short-fragment nucleoprotein (N) gene products (165 bp) (21). We used the PrimeScript one-step RT-PCR kit version 2 with a modified protocol (TaKaRa Bio, Inc., Shiga, Japan). The reaction mixture (22.5 μL) contained 7 μL of double-distilled water (ddH_2_O), 12.5 μL of one-step buffer, 1.25 μL of LN34 primers, 0.5 μL of PrimeScript one-step enzyme mix, and 2.5 μL of the RNA template. Reactions were performed using the Veriti thermal cycler (Applied Biosystems, USA) under the following conditions: 50°C for 30 min; 94°C for 2 min; 40 cycles of 94°C for 15 s, 50°C for 30 s, and 68°C for 2 min; and a final extension step at 68°C for 5 min. PCR products were visualized on a 1.5% agarose gel using Biotium gel green nucleic acid stain. A positive PCR result was expected to be observed as an amplicon of 165 bp (see Table S1 in the supplemental material).

### RT-PCR assay with a P1-304 primer set for phylogenetic analysis.

Second, we used the P1-304 primer set to amplify the long fragment (1,506 bp) of the entire N gene (22, 23). We used the TaKaRa PrimeScript one-step RT-PCR kit, and the volume for the master mix and the RNA template was the same as that used in the above-described procedures for the LN34 RT-PCR assay; a 1.5% agarose gel was used to visualize the positive result with an expected size of 1,506 bp. Before subjecting the samples to sequencing, specific bands were extracted and purified using the QIAquick gel extraction kit (Qiagen, Valencia, CA, USA). All RT-PCR products with successful amplification were sent to Macrogen, Inc., Philippines, for Sanger sequencing in both the forward and reverse directions. In addition to the above-mentioned primer sets, a cocktail of JW6 DPL (Duvenhage virus, rabies virus strain Pasteur and Lagos bat virus), JW6 M (Mokola virus), and JW6 E (European bat lyssavirus-1 and -2) primers was used for sequencing (see Table S1 in the supplemental material) (24).

### Phylogenetic analysis.

Original sequences were trimmed and edited using GeneStudio version 2.2.0.0 (http://genestudio.sharewarejunction.com/). Next, the trimmed sequences were aligned to the reference sequence (GenBank accession no. NC_001542.1). Multiple-sequence alignment of the N sequences was performed using Molecular Evolutionary Genetics Analysis version 11 (MEGA11) (http://www.megasoftware.net/). The phylogenetic tree was constructed using the maximum likelihood method with a bootstrap probability calculated from 500 replicates. In the analysis, the Tamura 3-parameter with gamma distribution model was selected as the substitution model based on the Akaike information criterion with a correction value in the model selection feature of the MEGA11 program. The cutoff value for the condensed tree was set at 70%. Other reference sequences used in the phylogenetic analysis were obtained from GenBank (https://www.ncbi.nlm.nih.gov/search/) (see Table S2 in the supplemental material). The sequences were then compared to those of other rabies lyssaviruses in the Philippines and Asia and lyssaviruses from elsewhere.

### Data analysis.

Background information for the sample was entered into a system (REDcap Consortium, Nashville, TN, USA). Statistical analyses were performed using Stata software (version 17; StataCorp, College Station, TX). The 95% confidence intervals (CIs) of sensitivity and specificity were calculated according to the methodology described previously by Hess et al. (25). To identify the statistical differences in the sensitivities and specificities of RT-PCRs using LFDs stored at refrigerated and room temperatures, we used McNemar’s chi-square test. A *P* value of <0.05 was considered significant. We set the minimum sample size of group 1 and group 2 to 124 samples to detect a >20% difference in sensitivity. Since the sensitivity of LN34 RT-PCR is nearly 100%, we considered that a reduction in sensitivity of more than 20% is practically insufficient for a rabies diagnostic test (13, 21).

### Data availability.

All nucleotide sequences obtained in this study are available in the DNA Data Bank of Japan (DDBJ) (http://getentry.ddbj.nig.ac.jp/top-j.html) (accession no. LC752950 to LC752983).

## RESULTS

Between 1 September 2020 and 14 May 2021, brain samples from 173 animals suspected of having rabies were submitted to the laboratory. Three samples were excluded because a DFAT was not performed since brain tissue was decomposed. Among the 170 enrolled individuals, 157 were dogs, 12 were cats, and 1 was a lagomorph (Table 1). In the first experiment, we stored 71 LFDs after being used for diagnosis in a refrigerator, including 26 DFAT-positive and 45 DFAT-negative samples (group 1). The median duration from the date of the LFD test until RNA extraction was 38 days (range, 5 to 112 days) (Table 1).

**TABLE 1:** Characteristics, rabies test results, and storage durations for 170 samples suspected of being positive for rabies according to experimental groups

Parameter	Value for group(s)			
	Total (*n* = 170)	1st expt		2nd expt, groups 3 and 4 (*n* = 35)
		Group 1 (*n* = 71)	Group 2 (*n* = 64)	
No. (%) of samples				
Species				
Dog	157 (92.4)	66 (93.0)	60 (93.8)	31 (88.6)
Cat	12 (7.1)	5 (7.0)	4 (6.3)	3 (8.6)
Other	1 (0.6)	0 (0)	0 (0)	1 (2.9)^*a*^
DFAT result				
Positive	92 (54.1)	26 (36.6)	43 (67.2)	23 (65.7)
Negative	78 (45.9)	45 (63.4)	21 (32.8)	12 (34.3)
LFD test result on-site				
Positive	92 (54.1)	26 (36.6)	43 (67.2)	23 (65.7)
Negative	78 (45.9)	45 (63.4)	21 (32.8)	12 (34.3)
Duration of LFD storage after the test (days)				
0–30	53 (31.2)	33 (46.5)	17 (26.6)	3 (8.6)
3–60	67 (39.4)	13 (18.3)	34 (53.1)	20 (57.1)
61–90	40 (23.5)	15 (21.1)	13 (20.3)	12 (34.3)
91–120	10 (5.9)	10 (14.1)	0 (0)	0 (0)

Median duration of LFD storage (days) (range)	43.5 (5, 112)	38 (5, 112)	46 (8, 83)	53 (29, 83)

a Lagomorph.

Among the 71 samples, 25 were positive and 46 were negative by the LN34 RT-PCR assay, with a sensitivity of 96.2% (95% CI, 80.4 to 99.9%) and a specificity of 100% (95% CI, 92.1 to 100%) (Table 2). Among the 25 positive samples, all samples were positive by RT-PCR with the P1-304 primer set (sensitivity, 100% [95% CI, 64.7 to 100%]). We obtained sequence data and constructed a phylogenetic tree from the positive samples amplified by the sequencing primers. Subsequently, we collected 64 LFDs, including 43 DFAT-positive and 21 DFAT-negative samples, and stored these at room temperature (group 2). The median duration of storage was 46 days (range, 8 to 83 days). The LN34 RT-PCR assay using LFDs stored at room temperature showed 100% sensitivity and 100% specificity. However, among the 43 samples that were positive using LN34 RT-PCR, P1-304 RT-PCR detected only 9 positive samples (sensitivity, 20.9% [95% CI, 9.6 to 39.7%]). The PCR products of the nine samples were used for subsequent genome sequence analysis. In the second experiment, samples from a single brain were applied to two LFDs and stored at 2°C to 8°C or room temperature. We used 35 samples, including 23 DFAT-positive and 12 DFAT-negative samples, and applied these to two LFDs each (group 3 and group 4). The results of LN34 RT-PCR were the same between the two groups. However, the sensitivity of the P1-304 RT-PCR was significantly higher for LFDs stored in a refrigerator than for those stored at room temperature (100% versus 4.5%; *P* < 0.01).

**TABLE 2:** Sensitivities and specificities of conventional LN34 RT-PCR and P1-304 RT-PCR using RNA extracted from LFDs according to storage conditions^*a*^

Parameter	Value for group							
	Group 1 (2°C–8°C) (*n* = 71)		Group 2 (room temp) (*n* = 64)		Group 3 (2°C–8°C) (*n* = 35)		Group 4 (room temp) (*n* = 35)	
	DFAT positive	DFAT negative	DFAT positive	DFAT negative	DFAT positive	DFAT negative	DFAT positive	DFAT negative
Conventional LN34 RT-PCR (amplicon of 165 bp)								
No. of positive samples	25	0	43	0	22	0	22	0
No. of negative samples	1	45	0	21	1	12	1	12
Sensitivity (%) (95% CI)	96.2 (80.4–99.9)		100 (91.8–100)		95.7 (78.1–99.9)		95.7 (78.1–99.9)	
Specificity (%) (95% CI)	100 (92.1–100)		100 (83.9–100)		100 (73.5–100)		100 (73.5–100)	
P1-304 conventional RT-PCR (amplicon of 1,506 bp)								
No. of positive samples	25		9		22		1	
No. of negative samples	0		34		0		21	
Sensitivity (%) (95% CI)	100 (64.7–100)		20.9 (9.6–39.7)		100 (62.7–100)		4.5 (0.1–25.3)	

a DFAT, direct fluorescent-antibody test; CI, confidence interval.

### Phylogenetic analysis.

**FIG 3:**
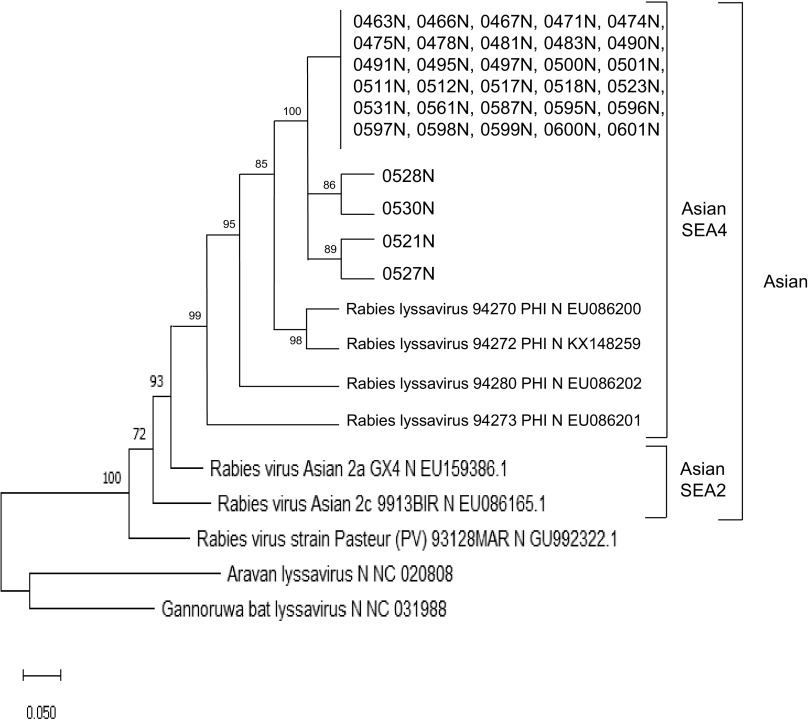
Phylogenetic tree of the 34 samples compared with other lyssaviruses constructed using the complete N gene (1,353 nucleotides [nt]).

Phylogenetic analysis was performed for 34 positive samples amplified with the sequencing primer among the 135 samples in groups 1 and 2. All acquired sequences had a very close resemblance to other strains in the Philippines. Phylogenetic analysis showed that all 34 sequences belonged to the Asian clade and were grouped into the Southeast Asia 4 (SEA4) subclade (Fig. 3).

## DISCUSSION

Our results indicated that used LFD strips can be utilized for the molecular analysis of rabies virus. In our study, while the LN34 RT-PCR assay amplifying short fragments with viral RNA extracted from LFDs showed high sensitivity and specificity regardless of the storage temperature, the sensitivities of RT-PCR using P1-304 RT-PCR amplifying long fragments for subsequent sequencing and phylogenetic analysis were significantly reduced in the LFDs stored at room temperature compared to the refrigerated kits. While the techniques described in this study are beneficial for the molecular analysis of samples from remote areas, they are not suitable for routine rabies diagnosis.

We showed that temperature control during the storage of the used LFDs is an important factor in the success rates of RT-PCR amplifying large fragments. This could occur due to the degradation of RNA on the LFD strips during storage. The degraded RNA fragments may not be long enough for RT-PCR assays aimed at generating long nucleic acid fragments but may be adequate for real-time RT-PCR or conventional RT-PCR amplifying small fragments. In our study, the amplicon produced by the P1-304 primer set was much larger than that produced by the LN34 primer set (1,506 bp versus 165 bp). A previous study by Eggerbauer et al. also showed that while they detected all 30 positive cases using a real-time RT-PCR assay, partial sequencing of the N gene (606 bp) was successful for only 2 of 5 samples when the LFDs were stored for 6 weeks at room temperature (17). A similar finding was observed in a rabies study using RNA extracted from a filter paper card. Importantly, while the primer set for 964 bp did not detect any positive samples, the primer set for the 290-bp N gene detected seven cases among nine brain samples tested (5). A study on malaria found that when used LFDs were applied to molecular analysis, the size of the PCR product similarly affected the success rate (9). A study on hepatitis C virus demonstrated that virus loads on filter paper were reduced when stored at room temperature compared to those kept at −20°C (26). In contrast, one study on foot-and-mouth disease virus observed no effect of storage conditions on virus recovery from LFDs when using a primer set to amplify the long fragment (1,156-bp or 1,135-bp product) (11). The storage temperature in our study might have been higher than the ones used in other studies owing to the tropical climate.

We successfully constructed a phylogenetic tree with all positive samples using sequencing primers (*n* = 34) for groups 1 and 2. In this study, phylogenetic analysis of the N gene revealed that all sequences were grouped into the Asian clade and the SEA4 subclade (27). We performed N gene sequencing for phylogenetic analysis in this study since N gene analysis is essential for classifying the lineage (27). The virus subclades are identical because this is a single-institute analysis using samples from one region. This analysis could be useful if the LFDs are collected from various sites. For further phylogenetic analysis, G and P genes should be used because they exhibit higher genetic diversity than N genes (28).

According to the World Health Organization rabies laboratory manual, rabies virus can be inactivated after 2 h of storage at room temperature on filter paper and shipped via regular mail routes (19, 20). This was based on the results of a cell inoculation test by Picard-Meyer et al. (7). That study showed that rabies virus on filter papers was inactivated after incubation at 25°C for 2 h (7, 19, 20). Consistent results were observed in studies on other viruses such as avian influenza and Newcastle disease viruses using filter paper (2, 4). In contrast, virus cultivation was observed in three of five filter papers after 17 h of storage after the application of brain tissue (6). Eggerbauer et al. evaluated the potential biohazard risks of six commercially available rabies LFDs. Among them, five LFDs were inactivated by 1 h of storage at room temperature or in the buffer solution in the kit (17). We also performed a rabies tissue culture infection test with the street rabies virus strain 1088 at a biosafety level 3 laboratory at Oita University, Japan (19, 20, 29) (data not shown). We applied a suspension of the homogenate of mouse brain tissue infected with rabies virus to the LFDs. The LFDs were stored for three different durations, 0.5, 2, or 4 h, at room temperature maintained at 25°C in the laboratory. Rabies virus was isolated by cell culture from all test strips stored for 0.5, 2, and 4 h. Our findings were limited due to the small sample size and need further evaluation. As there was insufficient evidence to rule out the biohazard risk of positive LFDs, further evaluation is necessary to optimize the assay buffer and the duration of storage at room temperature to inactivate the virus on the strip.

There are several limitations to our study. We did not evaluate some conditions related to the success rate of RT-PCRs. Future studies should investigate the optimum RNA extraction methods and the length of time for elution from the positive LFD strips. We did not evaluate the association between the storage durations and the success rates of RT-PCR for sequencing because the storage durations were relatively similar among the collected samples. Rasolonjatovo et al. could detect rabies virus by RT-PCR after 2 years of storage of the filter papers, although they did not perform sequencing using these samples (6). Guirou et al. found that the long-term storage of LFDs negatively affected the success rate of molecular detection of malaria-positive LFDs (10). We did not evaluate the RT-PCR assay generating midsize PCR products (between 165 bp and 1,506 bp). Future studies should evaluate the necessity for refrigerated storage if RT-PCR amplifying midsize fragments is used. Humidity should also be assessed, as one study on HIV using filter paper showed the importance of ensuring RNA stability (30). We did not use RT-qPCR to determine the viral loads; instead, we used conventional PCR to identify the RNA on the test strip. An RT-PCR protocol modified from the one in the manufacturer’s manual was used in this study. When we employed the modified procedure instead of the company’s protocol, there were no reduced sensitivities in the preliminary analysis.

In conclusion, used LFDs are a promising resource for subsequent molecular analysis of rabies virus. When an RT-PCR assay is employed to amplify large fragments for genome sequencing and phylogenetic analyses, refrigerated storage of the used LFDs is optimal.
