# Supplementary material for: Molecular Analysis of Rabies Virus Using RNA Extracted from Used Lateral Flow Devices
Source: J Clin Microbiol. 2023 Feb 22;61(3):e01543-22. doi: 10.1128/jcm.01543-22 (PMC10035306; doi:10.1128/jcm.01543-22)

Supplementary File 1

Figure S1. Flow of the procedure of elution of RNA from lateral flow device (LFD), RT-PCR and sequencing.

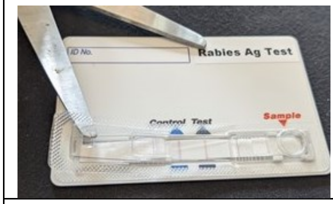	Step1.<br>Remove a plastic cover of the test strips from LFD.
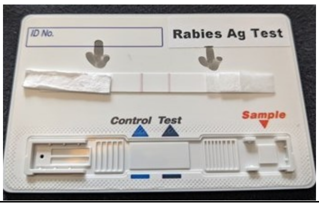	Step 2.<br>Cut the strips. Two parts of the absorbent paper are used for RNA extraction.
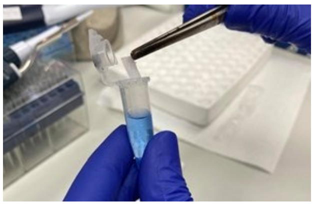	Step 3.<br>Put these two parts of absorbent paper into a tube with 1000 μL of ISOGEN II (NIPPON GENE CO., LTD) and incubate at room temperature for 1hour.<br>Perform subsequent procedures of RNAs extraction following the manufacturer’s procedure.
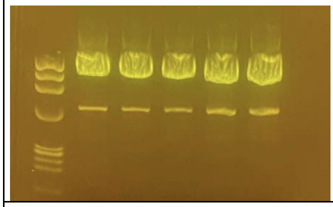	Step4.<br>Perform conventional RT-PCR with the p1 , 304 primer set, gel electrophoresis and gel purification.
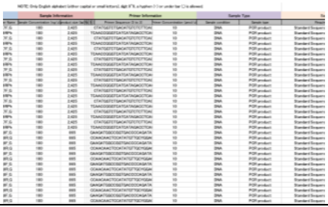	Step5.<br>Sequencing
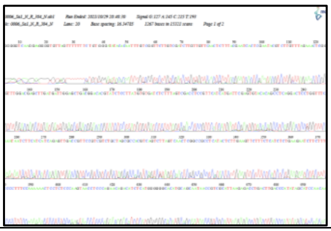	Step6.<br>Analyze the sequence data.

Table S1. List of primers used in this study.

Primer	Sequence (5'-3')	Position
LN34-F1 (Forward) [1]	ACG CTT AAC AAC CAG ATC AAA GAA	1-24
LN34-F2 (Forward) [1]	ACG CTT AAC AAC AAA ATC ADA GAA G	1-25
LN34-R (Reverse) [1]	CMG GGT AYT TRT AYT CAT AYT GRT C	140-164
P1 (Forward) [2]	ACA GAC AGC GTC AAT TGC AAA GC	28-50
304 (Reverse) [3]	TTG ACG AAG ATC TTG CTC AT	1515-1534
JW6 DPL (Duvenhage virus PV and Lagos bat virus) [4]	CAA TTC GCA CAC ATT TTG TG	660-641
JW6 M (Mokola virus) [4]	CAG TTA GCG CAC ATC TTA TG	660-641
JW6 E (EBLs 1 and 2)[4]	CAG TTG GCA CAC ATC TTG TG	660-641

1. Wadhwa A, Wilkins K, Gao J, Condori Condori RE, Gigante CM, Zhao H, et al. A Pan-Lyssavirus Taqman Real-Time RT-PCR Assay for the Detection of Highly Variable Rabies virus and Other Lyssaviruses. PLoS Negl Trop Dis. 2017;11: e0005258. doi:10.1371/journal.pntd.0005258

2. Cai, L., Tao, X., Liu, Y., Zhang, H., Gao, L., Hu, S., Liu, F., Li, H., Shen, X., Liu, J., Wang, S., & Tang, Q. (2011). Molecular characteristics and phylogenetic analysis of N gene of human derived rabies virus. Biomedical and environmental sciences : BES, 24(4), 431–437. https://doi.org/10.3967/0895-3988.2011.04.015

3. Orciari, L. A., Niezgoda, M., Hanlon, C. A., Shaddock, J. H., Sanderlin, D. W., Yager, P. A., & Rupprecht, C. E. (2001). Rapid clearance of SAG-2 rabies virus from dogs after oral vaccination. Vaccine, 19(31), 4511–4518. https://doi.org/10.1016/s0264-410x(01)00186-4

4. Heaton PR, Johnstone P, McElhinney LM, Cowley R, O'Sullivan E, Whitby JE. Heminested PCR assay for detection of six genotypes of rabies and rabies-related viruses. J Clin Microbiol. 1997;35: 2762–2766. doi:10.1128/jcm.35.11.2762-2766.1997

Table S2. List of Lyssaviruses samples used in the phylogenetic analysis (Figure 3)

Country	Identification number/ Strain	Cluster	Host	Year	Gene	Accession No.
Philippines	94270 PHI	Asian SEA4	Dog	1994	N	EU086200
Philippines	94272 PHI	Asian SEA4	Dog	1994	N	KX148259
Philippines	94273 PHI	Asian SEA4	Dog	1994	N	EU086201
Philippines	94280 PHI	Asian SEA4	Dog	1994	N	EU086202
China	RV/GX4/1994	Asian 2a	Dog	1994	N	EU159386
Myanmar	RV/9913BIR/1999	Asian 2c	Dog	1999	N	EU086165
Morroco	93128MAR	Rabies virus strain Pasteur (PV)	-	1993	N	GU992322
Kyrgyzstan	Aravan Lyssavirus		Bat	2003	N	NC020808
Sri Lanka	Gannoruwa Bat Lyssavirus		Bat	2016	N	NC031988

These reference sequences used were obtained from the GenBank database (https://www.ncbi.nlm.nih.gov/search/).

Supplementary file 2. STARD (Standards for Reporting of Diagnostic Accuracy) checklist and diagram

Title "Molecular analysis of the rabies virus using RNA extracted from used lateral flow devices and the optimum storage conditions"

Section & Topic	No	Item	Reported on page #
**TITLE OR ABSTRACT**			
	**1**	Identification as a study of diagnostic accuracy using at least one measure of accuracy (such as sensitivity, specificity, predictive values, or AUC)	Page 1
**ABSTRACT**			
	**2**	Structured summary of study design, methods, results, and conclusions (for specific guidance, see STARD for Abstracts)	Page 1-2, Abstract
**INTRODUCTION**			
	**3**	Scientific and clinical background, including the intended use and clinical role of the index test	Page 2-4
	**4**	Study objectives and hypotheses	Page 4
**METHODS**			
Study design	**5**	Whether data collection was planned before the index test and reference standard were performed (prospective study) or after (retrospective study)	Reference 5,8,9
Participants	**6**	Eligibility criteria	Reference 8,9
	**7**	On what basis potentially eligible participants were identified (such as symptoms, results from previous tests, inclusion in registry)	Reference 8,9
	**8**	Where and when potentially eligible participants were identified (setting, location and dates)	Page 4, 6
	**9**	Whether participants formed a consecutive, random or convenience series	Page 4, Reference 8,9
Test methods	**10a**	Index test, in sufficient detail to allow replication	Page 5, S1 figure, S2 file
	**10b**	Reference standard, in sufficient detail to allow replication	Page 4, Reference 8,9
	**11**	Rationale for choosing the reference standard (if alternatives exist)	NA
	**12a**	Definition of and rationale for test positivity cut-offs or result categories of the index test, distinguishing pre-specified from exploratory	Page 5, S2 file
	**12b**	Definition of and rationale for test positivity cut-offs or result categories of the reference standard, distinguishing pre-specified from exploratory	Page 4, Reference 8,9

	**13a**	Whether clinical information and reference standard results were available to the performers/readers of the index test	Page 4
	**13b**	Whether clinical information and index test results were available to the assessors of the reference standard	Page 4
Analysis	**14**	Methods for estimating or comparing measures of diagnostic accuracy	Page 5
	**15**	How indeterminate index test or reference standard results were handled	NA
	**16**	How missing data on the index test and reference standard were handled	NA, no missing data
	**17**	Any analyses of variability in diagnostic accuracy, distinguishing pre-specified from exploratory	NA
	**18**	Intended sample size and how it was determined	NA
**RESULTS**			
Participants	**19**	Flow of participants, using a diagram	STARD checklist
	**20**	Baseline demographic and clinical characteristics of participants	Table 1
	**21a**	Distribution of severity of disease in those with the target condition	NA
	**21b**	Distribution of alternative diagnoses in those without the target condition	NA
	**22**	Time interval and any clinical interventions between index test and reference standard	Table 1
Test results	**23**	Cross tabulation of the index test results (or their distribution) by the results of the reference standard	Table 2
	**24**	Estimates of diagnostic accuracy and their precision (such as 95% confidence intervals)	Table 2
	**25**	Any adverse events from performing the index test or the reference standard	NA
**DISCUSSION**			
	**26**	Study limitations, including sources of potential bias, statistical uncertainty, and generalisability	Page 11
	**27**	Implications for practice, including the intended use and clinical role of the index test	Page 9-11
**OTHER INFORMATION**			
	**28**	Registration number and name of registry	NA
	**29**	Where the full study protocol can be accessed	S2 file
	**30**	Sources of funding and other support; role of funders	Page 11

Abbreviation: NA, not applicable; LFD, lateral flow device; dFAT, direct fluorescent antibody test

STARD Diagram for LN34 RT-PCR

*= 35 samples were subjected to two kits each (one stored in room temperature, one stored in ref temperature)

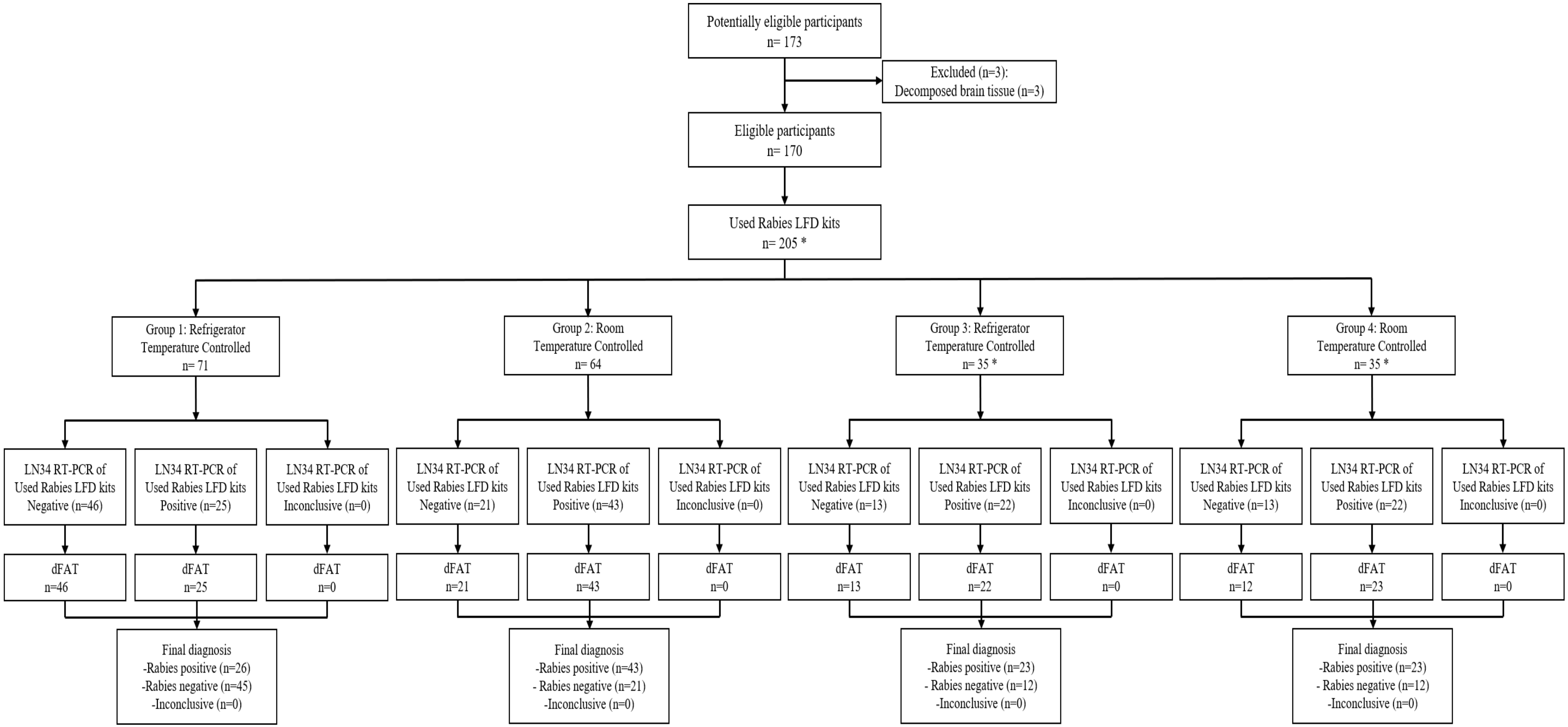

STARD Diagram for P1-304 RT-PCR

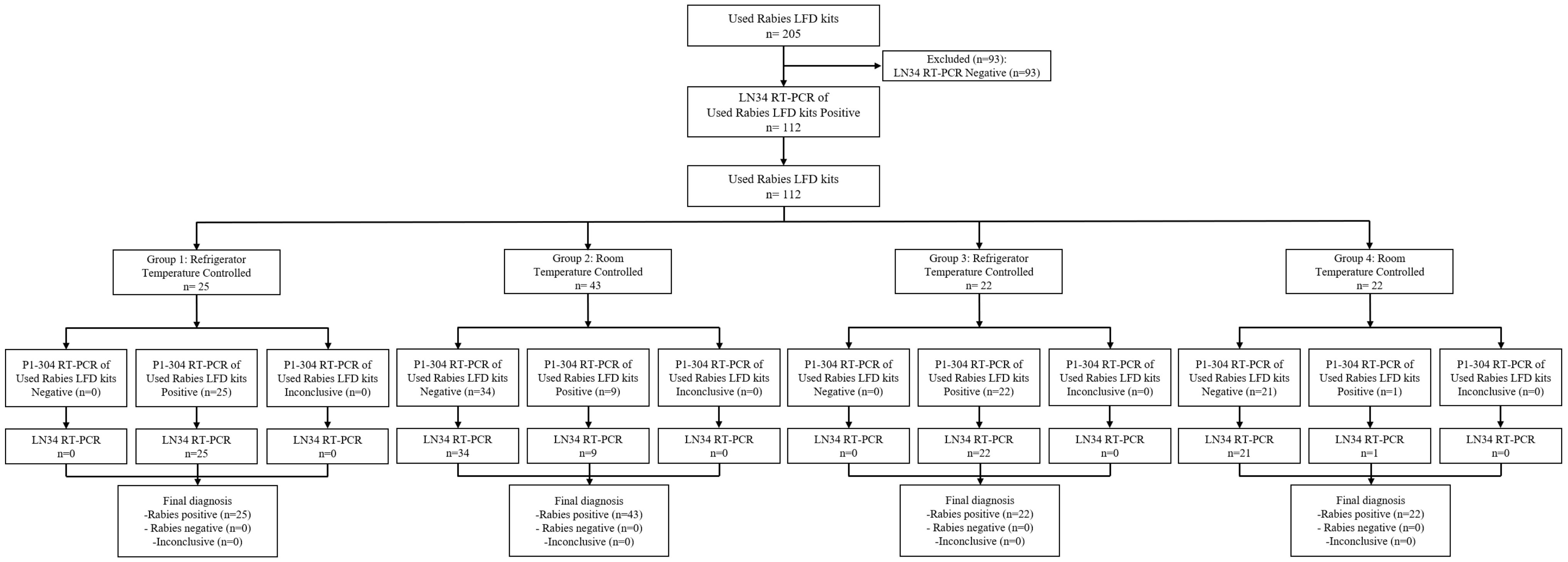

Supplement: Supplemental file 1 — Supplemental material. Download jcm.01543-22-s0001.pdf, PDF file, 0.6 MB [file jcm.01543-22-s0001.pdf]
